# Proteomic profiling of human dental enamel affected by molar incisor hypomineralisation of different clinical severity grades: an in vitro study

**DOI:** 10.1007/s40368-024-00911-9

**Published:** 2024-06-06

**Authors:** F. Rexhaj, N. Sabel, A. Robertson, T. Lundgren

**Affiliations:** Department of Pediatric Dentistry, Institute of Odontology at the Sahlgrenska Academy, University of Gothenburg, P. O. Box 450, 40530 Göteborg, Sweden

**Keywords:** Human, Dental enamel, Molar incisor hypomineralization, Liquid chromatography–mass spectrometry, Proteomics, Children

## Abstract

**Purpose:**

The aim of this study was to explore the potential to profile and distinguish varying clinical severity grades of MIH, compared to normal enamel, using proteomics.

**Methods:**

Liquid chromatography–mass spectrometry analyses were conducted on enamel samples of extracted teeth, from 11 children and adolescents, spanning an age range of 6–18 years. Enamel powder samples were collected from extracted, third molars (*n* = 3) and first permanent molars diagnosed with MIH (*n* = 8). The MIH tooth samples were categorized into subgroups based on clinical severity grade. The data were statistically analyzed using ANOVA and Welch’s *t* test.

**Results:**

Teeth affected by MIH exhibited a diverse array of proteins, each with different functions related to dental enamel, distinguishing them from their normal enamel counterparts. The application of microdissection combined with LC–MS techniques has revealed the potential to discern unique proteomic profiles among MIH-affected teeth, characterized by varying clinical severity grades. Both analyzed MIH groups displayed consistent trends in the presentation of biological processes, including underabundance of proteins primarily associated with cell organization and biogenesis. Furthermore, proteins linked to cell death were overabundant in both MIH groups.

**Conclusion:**

Proteomics enabled the detection and differentiation of various proteins across different clinical severity grades of MIH.

**Supplementary Information:**

The online version contains supplementary material available at 10.1007/s40368-024-00911-9.

## Introduction

Molar Incisor Hypomineralization (MIH) is used to describe a wide-ranging condition, characterized by demarcated opacities on the first permanent molars, often simultaneously affecting the permanent incisors. The globally pooled prevalence is estimated to be 14.2% (Zhao et al. 2018). In MIH, both the grades, surface extent, and color on the dental crown vary between individuals, and even among teeth in the same individual. Clinical appearances range from white demarcated opacities, with preserved morphology to cases with yellow–brown discolorations, with or without morphology preserved (Ghanim et al. 2017). Despite the diversity of clinical manifestations and subjective symptoms, previous proteomic studies have assessed MIH as an entity, or profiled based on enamel color appearance, in previous proteomic studies (Farah et al. 2010; Mukthar et al. 2022). The etiology for this condition remains unknown.

In the field of proteomic analysis, numerous research studies have investigated enamel proteins using various methods, including amino acid analysis, sodium dodecyl sulfate–polyacrylamide gel electrophoresis (SDS-PAGE), matrix-assisted laser desorption/ionization time-of-flight/time-of-flight mass spectrometry (MALDI-TOF/TOF MS), and protein sequencing. These studies have explored both normal and pathological enamel, such as conditions like amelogenesis imperfecta, dental fluorosis and molar incisor hypomineralization (Wright et al. 1989, 1992, 1996, 1997; Takagi et al. 1998; Açil et al. 2005; Nielsen-Marsh et al. 2009; Mangum et al. 2010; Farah et al. 2010).

LC–MS, as an advanced technology, applied on erupted, permanent, human enamel, has helped us in a transformative era by facilitating quantitative analysis and enabling a broader spectrum of protein detection in human dental enamel compared to other methods e.g. SDS-PAGE (Castiblanco et al. 2015; Jagr et al. 2019; Mukthar et al. 2022; Rexhaj et al. 2023). Through LC–MS, intact proteins from hard tissue undergo enzymatic digestion by proteolysis, into smaller peptides. The enamel peptides are then separated and analyzed by LC–MS (Wolters et al. 2001; Porto et al. 2011).

Numerous proteins play a role in the developing process of dental enamel matrix. In mature dental enamel, most of these proteins have been degraded, leaving a few remnants of residual organic components. The organic content in MIH enamel, mainly proteins, is confirmed to be higher, compared to normal enamel (Farah et al. 2010; Mukthar et al. 2022). Recent advancements in this research field have revealed a variety of enamel specific and enamel non-specific proteins present in healthy and MIH-affected human enamel. In the study by Mukthar et al. (2022), an upregulation of proteins in healthy enamel was observed, including collagens, α1-anti-trypsin, kallikrein-4 (KLK4), matrix metalloprotease-20 (MMP-20), alpha-2-macroglobulin, and alpha-2-HS-glycoprotein. In contrast, enamel affected by MIH exhibited an overexpression of albumin, calcium-binding proteins, anti-thrombin III, dentin sialophosphoprotein (DSPP), and proteins associated with stress response and inflammatory biological functions. Farah et al. (2010) identified proteins across all enamel types, such as serum albumin, type I collagen, and anti-trypsin. In MIH enamel, referred to as yellow and brown enamel, the presence of serum anti-thrombin was noted. Furthermore, Jagr et al. (2019) focused on proteins at the dentinoenamel junction (DEJ), highlighting collagens as predominant. Their findings showed enamel specific proteins like ameloblastin and amelogenin, shedding light on biological functions, such as calcium ion-binding, extracellular matrix formation, cytoskeleton organization, cytoskeletal protein binding, cell adhesion, and transport.

In particular, it is crucial to acknowledge the limited number of studies in the existing literature that have explored healthy human permanent enamel using LC–MS, each employing slightly varying methodologies (Castiblanco et al. 2015; Jagr et al. 2019; Rexhaj et al. 2023). To the best of available knowledge, Mukthar et al. (2022) is the only study to undertake a comparative analysis of healthy permanent enamel with MIH using LC–MS. Consequently, there exists a significant gap in the current body of knowledge within this research domain. Varying clinical severity grades of MIH have not been comprehensively assessed using proteomics. Therefore, the aim of this study was to explain the possibility to profile and distinguish between varying clinical severity grades of MIH using proteomics.

## Materials and methods

### Patients and tooth samples

Normal third molars (*n* = 3) and MIH-affected first molars (*n* = 8) were obtained from extracted teeth of 11 children and adolescents. Hypomineralized tooth samples were divided in two subgroups, according to the European Academy of Paediatric Dentistry’s (EAPD) scoring criteria for MIH (Ghanim et al. 2017). Group MIH I was identified as enamel with occlusal white/creamy demarcated opacities and group MIH II classified as yellow/brown demarcated opacities, respectively, without post-eruptive breakdown (Table 1).

**Table 1:** Description of assessed samples according to clinical severity grades

Group	Description	Sample No.
Controls	Healthy enamel with no clinical or radiographical sign of caries or mineralization disturbances	C1–C3
MIH I	White/creamy demarcated opacities, without post-eruptive breakdown	M1–M4
MIH II	Yellow/brown demarcated opacities, without post-eruptive breakdown	M5–M8

Hypomineralized tooth samples were divided according to the European Academy of Paediatric Dentistry, EAPD

To rule out the presence of caries, teeth underwent radiographic and clinical examinations. Prior to dental enamel microdissection and proteomic analyses, the teeth were examined using a Leica M80 microscope at × 2 magnification, and radiographically inspected with a conventional dental X-ray, Planmeca ProX, image plate (8 mA, 63 kV, 0.32 s). The inclusion criteria for healthy teeth were met if deemed normal and free from caries, fillings, or mineralization disturbances. The teeth were stored in 70 vol% ethanol directly after tooth extraction, until processing. To eliminate possible pellicle and periodontal ligament remnants, root surfaces were scraped off with a medium grade polishing disk for composite, under water cooling. Additionally, to remove surface debris, all tooth surfaces were etched with 35% phosphoric acid for 30 s and washed off with tap water.

### Microdissection of dental enamel

Dental enamel was dissected in accordance with the method described by Rexhaj et al 2023. Briefly, autoclaved equipment was used during the micro-dissection of dental enamel. Between each dental enamel microdissection preparation, tools, burs, and containers were changed. The outer part of hypo-mineralized dental enamel was removed using a high-speed North Bel Diamant 830/012 FG bur at maximum speed and water cooling, resulting in an enamel powder slurry collected in tube vials (Sarstedt, Nümbrecht, Germany). Twenty milliliters of the collected powder slurry from each tooth was centrifuged in a cooled centrifuge at 150 × g for 5 min, in order to collect enamel powder pellets. The supernatant was discarded, and the enamel powder pellet was stored at minus 80 °C in the vials, until protein analysis.

### Global relative quantification

Proteins were extracted from the enamel powder pellets and processed in accordance with the method described by Rexhaj et al 2023. Briefly, the filter-aided sample preparation (FASP) method was employed, modified from Wisniewski et al. (2009). Peptides were isobaric labeled using Tandem Mass Tag reagents (11-plex, Thermo Fischer Scientific) according to the manufacturer’s instructions. The samples were combined, and the TMT set was fractionated with high pH-reversed phase chromatography (pH 10), into 20 fractions. The TMT set was analyzed in an orbitrap Fusion™ Tribrid™ mass spectrometer, interfaced with an Easy-nLC1200 nanoflow liquid chromatography system (both Thermo Fisher Scientific) in Multinotch Mode, MS2 fragmentation for identification, and MS3 fragmentation for relative quantification, by comparing TMT reporter ion intensities (McAlister et al. 2014).

### Statistics and comparisons

A comparative analysis was conducted on normalized proteomic abundance data derived from two categories of enamel defects: white demarcated opacities (Group MIH I, *n* = 4) and discolored hypomineralizations (Group MIH II, *n* = 4). The analysis was carried out using ANOVA (*p* < 0.05) in R version 4.2.1 software, and the results were visually presented through a Principal Component Analysis (PCA) plot, to explore proteomic pattern variations across distinct clinical severity grades. Normalized abundances were utilized for global relative quantification. Proteins were filtered with a 5% FDR, with trypsin as the search enzyme.

Using *R* version 4.2.1, normalized proteomic abundance data from MIH samples were compared to normal enamel using Welch’s *t* test (*p* < 0.05) on log2-transformed values of MIH samples (Group MIH I, *n* = 4 and Group MIH II, *n* = 4), compared to mean average of controls (*n* = 3). To adjust for a centered scale around 0, ratios were calculated by dividing each sample’s normalized abundance values for MIH teeth by the mean abundance of the controls. From these calculations in *R*, significant proteins were plotted in heatmaps. Biological processes were classified according to the classification system used in the universal knowledge database UniProt (http://www.uniprot.org).

### Data analysis

Protein identification and quantification were performed, using Proteome Discoverer version 2.2 (Thermo Fisher Scientific) against the Swissprot Homo sapiens database, employing Mascot 2.5.1 (Matrix Science, London, United Kingdom) with a peptide tolerance of 5 ppm and a fragment ion tolerance of 0.6 Da. Peptides, accepted with zero missed cleavages, methionine oxidation as variable modification, alkylation and TMT reagent modification on lysine and peptide *N*-terminus, were set as fixed modifications. Trypsin was selected as the enzyme in the search. Percolator was used for PSM validation with a False Discovery Rate (FDR) threshold of 5%. Each sample was normalized based on the total peptide amount and only unique peptides were used for quantification. Quantified proteins were filtered with a 5% False Discovery Rate (FDR) at both the peptide and protein levels, and grouped by sharing the same sequences to minimize redundancy.

### Ethics

The Central Ethical Review Board, Gothenburg, Sweden, granted an ethical approval for this study (Dnr 924-16). The study was conducted in full accordance with ethical principles outlined in to the *World Medical Association Declaration of Helsinki*. Patients and parents were informed regarding the aim and study design. Written and informed consent from the patients and parents was obtained prior to the donation of extracted teeth.

## Results

### Proteomic profiling of varying clinical severity grades of hypomineralized enamel with principal component analysis

**Fig. 1:**
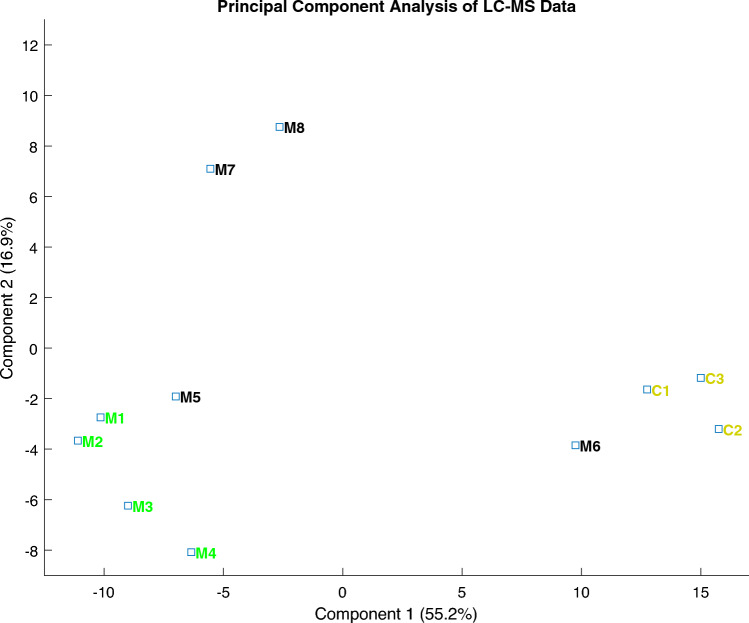
Principal component analysis plot illustrating division of MIH and normal enamel samples. 72% of the variance between MIH and normal enamel accounts for the first two components, i.e., enamel protein abundance data. Varying clinical severity grades of MIH (M1–M4 and M5–M8), compared to normal enamel (C1–C3), exhibit well-clustered appearances. White demarcated opacities (M1–M4) are well-clustered, showing a similar proteomic profile, well-distinguished from controls. Discolored hypo-mineralizations, without enamel breakdown (M5–M8), exhibit a broader range in proteomic profile and variance

Varying clinical severity grades of MIH, compared to healthy controls, display well-clustered appearances (Fig. 1). White demarcated opacities, with preserved morphology (Group MIH I), are well-clustered and show a similar proteomic profile, which is clearly distinguishable from the controls. Discolored hypo-mineralizations, with preserved morphology (Group MIH II), display a spread in proteomic profile and variance (Fig. 1). Proteins with statistically significant differences in abundance in MIH enamel, compared to normal enamel, were identified (ANOVA, *p* < 0.05). Using Principal Component Analysis, varying clinical severity grades of hypo-mineralized dental enamel could be differentiated (Fig. 1). 72.1% of the variance between hypo-mineralized and normal enamel is explained by the first two components, i.e., dental enamel protein abundance data.

### Proteomic comparison of white and discolored demarcated opacities with normal enamel

**Fig. 2:**
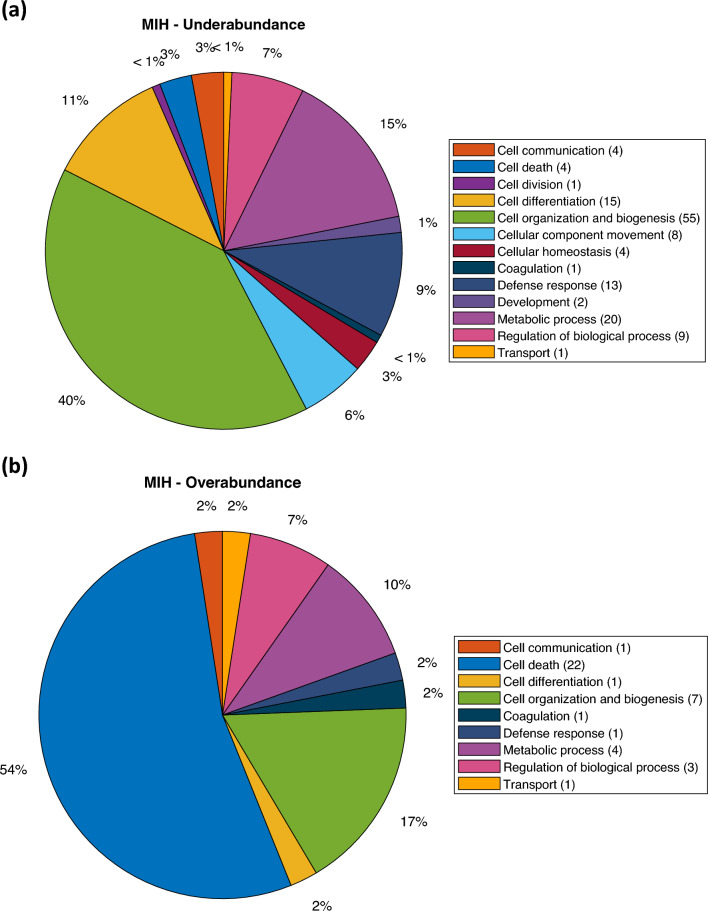
Circular diagrams illustrating the percentage distribution of proteins in combined MIH-I and MIH-II groups versus normal enamel. The prevailing biological processes among the underabundant proteins **a** primarily included cell organization and biogenesis (40%) as well as metabolic processes (15%). Conversely, the predominant biological process among the overabundant proteins **b** primarily comprised cell death (54%)

**Fig. 3:**
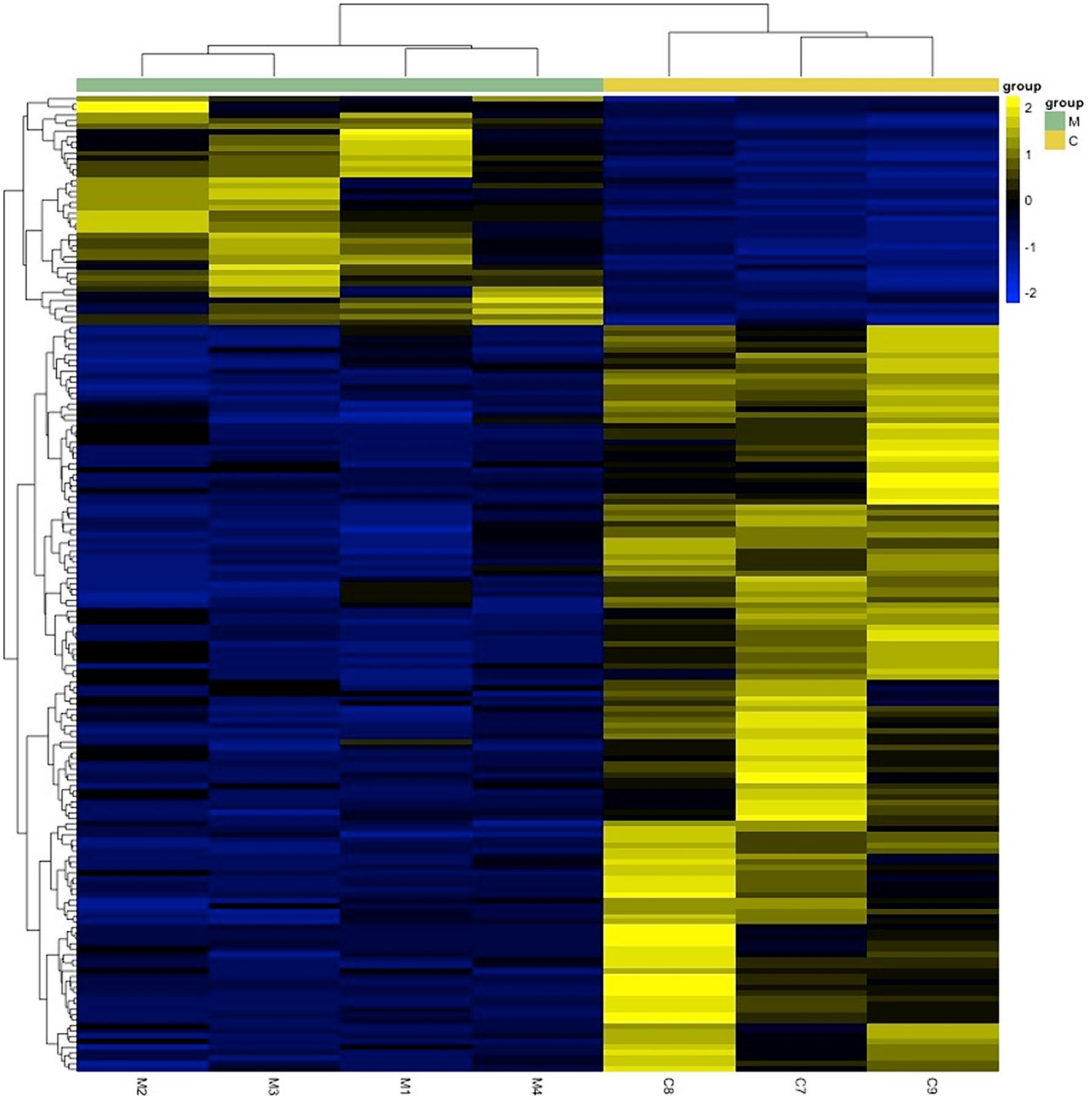
Heatmap showing overabundant (yellow) and underabundant (blue) proteins in group MIH I enamel (samples M1–M4), compared to normal enamel (samples C1–C3). Proteins with similar expressions are clustered (brackets, left row). Protein groups (M1–M4 and C1–C3) with similar expressions are adjacent (brackets, top). Proteins with over- and underabundances, 179 in total, were found (Supplementary data). In enamel opacities (M1–M4), 42 proteins were overabundant, mainly having cell death functions. Underabundance of proteins in the enamel opacity group mainly comprised cell organization and biogenesis proteins

**Fig. 4:**
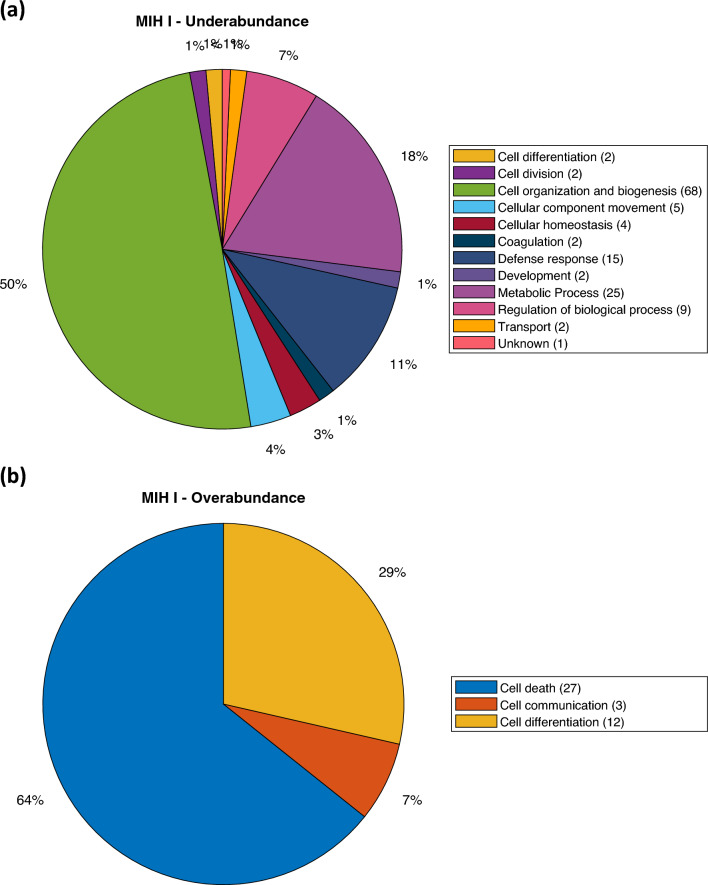
Circle diagrams showing distribution (%) of proteins in group MIH I, compared to normal enamel. The dominating biological processes among underabundant proteins **a** were mainly involved in cell organization and biogenesis (50%), metabolic process (18%), and defense response (11%). The dominating biological processes among overabundant proteins **b** were mainly cell death (64%)

**Fig. 5:**
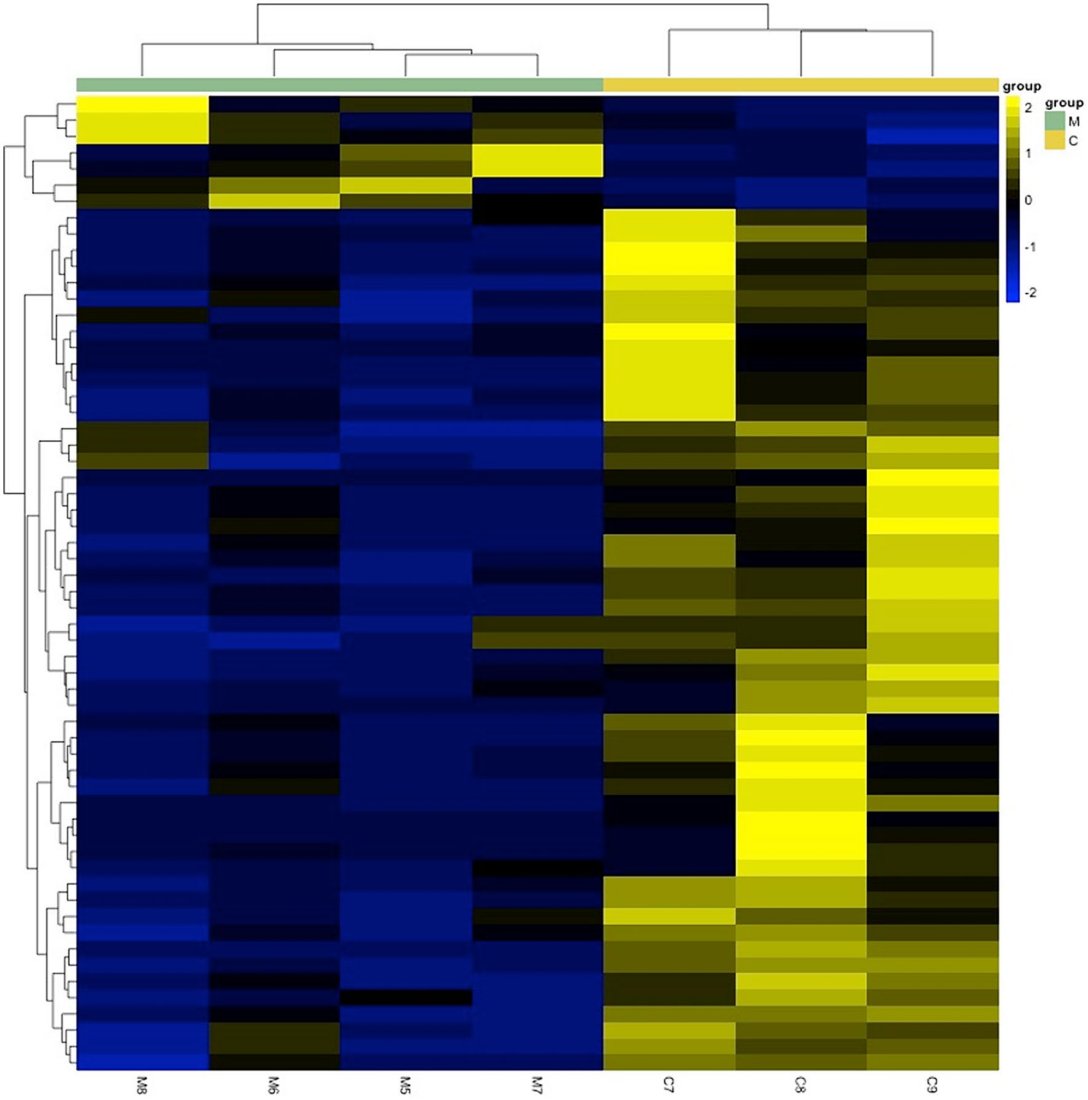
Heatmap illustrating overabundance (yellow) and underabundance (blue) proteins in group MIH II enamel (samples M5–M8), compared to normal enamel (samples C1–C3). Proteins with similar expressions are clustered (brackets, left row). Protein groups (M5–M8 and C1–C3) with similar expressions are adjacent (brackets, top). Proteins with over- and underabundances, 60 in total, were found. In enamel opacities (M5–M8), 7 proteins were overabundant, mainly having cell death functions. Underabundant proteins in the enamel opacity group mainly comprised of cell organization and biogenesis proteins, in addition to defense response (Supplementary data)

**Fig. 6:**
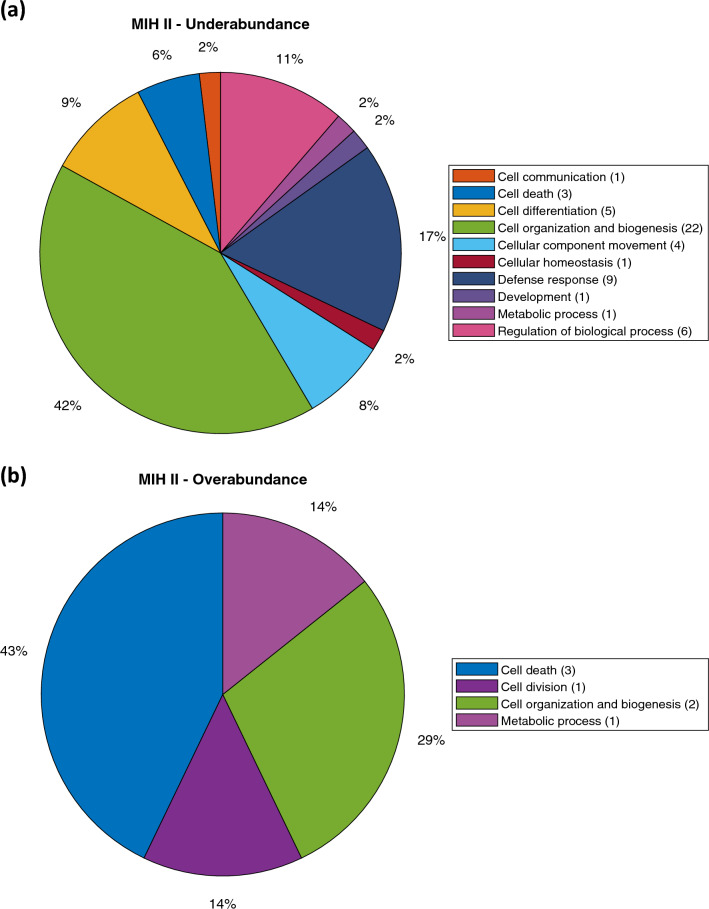
Circle diagrams showing distribution (%) of proteins in group MIH II, compared to normal enamel. The dominating biological processes among underabundant proteins **a** were mainly involved in cell organization and biogenesis (42%), defense response (17%) and regulation of biological process (11%). The dominating biological processes among overabundant proteins **b** were mainly cell death (43%)

A statistical analysis of MIH enamel as an entity revealed that 43 proteins were overabundant, while 137 proteins were underabundant compared to normal enamel (Supplementary data). The results indicate an overabundance of proteins related to cell death as the dominant biological process (Fig. 2b). The biological process showing underabundance primarily involves proteins related to cell organization and biogenesis (Fig. 2a). Additionally, a statistical analysis conducted separately for the MIH I and MIH II groups, instead of treating MIH enamel as a single entity, revealed several statistically different proteins between normal enamel compared to group MIH I and MIH II. In total, 179 proteins with varying levels of abundance were identified in group MIH I (Fig. 3). Among these, the enamel group MIH I exhibited 42 proteins that were overabundant, primarily associated with cell death functions (Fig. 4b). This overabundance was dominated by proteins, such as desmoplakin and keratins (Supplementary data), which may reflect impaired cell functions of ameloblasts during enamel formation or the trapping of proteins within porous, hypo-mineralized enamel. Underabundance of proteins in group MIH I was largely composed of cell organization and biogenesis proteins, e.g., hemoglobin subunit beta, complement C3, and fibrinogen (Fig. 4a). Normal enamel compared to group MIH II, displayed 60 over- and underabundant proteins (Fig. 5). Overabundant proteins (*n* = 7) essentially comprising biological processes of cell death, similar to those of MIH group I (Fig. 6b), mainly junction plakoglobin and keratin (Supplementary data). Underabundant proteins (*n* = 53) comprised biological processes, similar to data obtained from group MIH I (Fig. 6a).

Conducting a statistical analysis of MIH enamel as an entity, rather than separately analyzing the MIH I and MIH II groups, does not seem to significantly impact the outcome of underabundance and overabundance of biological processes on a broader scale. The results suggest that the dominant group remains cell death, while the biological process exhibiting underabundance still involves proteins related to cell organization and biogenesis.

## Discussion

A comparative analysis was conducted between normal enamel and MIH enamel. By employing a microdissection methodology combined with a LC–MS technique, the study reveals the potential to distinguish distinctive proteomic profiles within MIH enamel that display varying clinical severity grades. The proteomic profiles of controls and individuals in the MIH groups I and II exhibit a well-separable appearance.

Throughout the study, a quantitative analysis was conducted to identify proteins with statistically significant differences and their associated biological processes. To underscore these findings, the proteomic results are discussed in relation to previous LC–MS studies and incorporated with data from other research focusing on healthy enamel or MIH enamel from a proteomic perspective. In the field of proteomic analysis of hard tissues, particularly erupted permanent human enamel, the predominant focus has been on two fundamental techniques: LC–MS (Castiblanco et al. 2015; Jagr et al. 2019; Mukthar et al. 2022, Rexhaj et al. 2023) and SDS-PAGE (Açil et al. 2005; Farah et al. 2010). LC–MS, as a cutting-edge technology, has revolutionized the field by enabling quantitative analysis and the detection of a more extensive array of proteins compared to SDS-PAGE. Furthermore, it is essential to acknowledge the limited number of studies in the literature that have explored healthy permanent enamel using LC–MS, each employing slightly different methodologies (Jagr et al. 2019; Castiblanco et al. 2015; Rexhaj et al. 2023). As far as available knowledge extends, only Mukthar et al. (2022) conducted a comparative analysis of healthy permanent enamel with MIH through the application of LC–MS. Thus, this study bridges a critical gap in the current body of knowledge in this area of research.

Well-established animal models provide valuable information about the proteins present in the extracellular matrix during amelogenesis, both in the secretory and maturation phases. In addition to human permanent enamel, several studies have been conducted on animal models, including both erupted and non-erupted teeth. Rats and mice, with continuously growing maxillary and mandibular incisors, have become widely used animal models. This characteristic allows for the study of all stages of amelogenesis on a single incisor at any given time throughout the animal's lifespan (De Lima Leite et al. 2018). Pig teeth have also been utilized in dental research due to their availability, large size, and similarity in size and morphology to human teeth (Robinson et al. 1987; Fincham et al. 1999; Green et al. 2019; Gil-Bona and Bidlack 2020). Gil-Bona and Bidlack (2020) highlight the advantages of the porcine model for studying spatial changes within the mineralizing tooth crown and throughout the thickness of the enamel layer. The similarity between human and porcine teeth is particularly useful for modeling early stage enamel formation.

Following the maturation, cell death of ameloblast by apoptosis is a normal process during amelogenesis. Recent studies indicate that cell death during embryonic development generates several signaling molecules, which affect the behavior of adjacent cells, stimulating morphogenesis, cell migration, and alteration of cell fate during odontogenesis (Lacruz et al. 2017; Abramyan et al. 2021). MIH enamel expresses a diverse and irregular hypo-mineralization appearance pattern, indicating an impact on ameloblasts at a critical phase during amelogenesis (Sidaly et al. 2016). One might speculate that such an insult could cause an increase in protein remnants related to biological processes of cell death to be retained in the mature enamel.

Enamel development comprises several functional stages: the secretory and maturation stages, with additional subdivisions, such as presecretory, early secretory, late secretory, transition, preabsorptive, early maturation, and late maturation stages. Amelogenesis involves various activities, including the formation and subsequent removal of a proteinaceous matrix, ion transport, pH regulation, and apoptosis, resulting in avascular enamel devoid of regenerative properties (Lacruz et al. 2017; Nanci, 2008). Dental development extends beyond the mineralization of crown and root tissues, continuing until teeth reach functional occlusion postnatally. Several theories on tooth eruption exist, yet the regulatory mechanisms remain largely unknown (Wise et al. 2002). Tooth eruption occurs in three distinct stages: pre-eruptive tooth movement, eruptive tooth movement, and post-eruptive tooth movement. The second stage, eruptive tooth movement, involves the onset of root formation until the crown emerges into the oral cavity. This stage includes phases, such as intra- and supraosseous eruption, stages characterized by epithelial cell death within an inflammatory environment and bone resorption. One might speculate if proteins originating from both epithelial and inflammatory cells may persist within abnormally developed enamel, causing protein entrapment or incorporation during tooth eruption. This could potentially contribute to an elevated protein abundance in hypomineralized enamel (Kjaer, 2014; Richman 2019).

From a proteomic perspective, MIH differs from other enamel disturbances e.g., Amelogenesis Imperfecta (AI), which has shown increased residual abundance of amelogenin. The findings of this study, consistent with other research, indicate an elevated protein content in MIH enamel, but not an increased abundance of amelogenin. Serum proteins, including albumin, have been suggested as an inhibitor of apatite crystal growth (Farah et al. 2010; Mangum et al. 2010). MIH enamel is characterized by a high content of serum albumin, hemoglobin, alpha-1-anti-trypsin, anti-thrombin III, and serpin B3 (Mangum et al. 2010). Recent studies by Williams et al. (2020) and Perez et al. (2020) have proposed that proteins, such as albumin and potentially other blood-associated proteins, may act as predominant mineralization inhibitors in hypo-mineralized enamel. In the present study, serum albumin, alpha-1-anti-trypsin, Antithrombin III, and Serpin B3 were detected with no statistically significant difference observed between MIH and normal enamel. However, hemoglobin subunit gamma-2 showed an overabundance in MIH compared to normal enamel. Further investigation is warranted to explore the relationship between serum albumin, and the presence and activity of different protease inhibitors, such as anti-thrombin-III and alpha-1-anti-trypsin, in MIH enamel.

Intriguingly, the analyzed MIH groups exhibit similar trends in the presentation of biological processes, both in over- and underabundant proteins (Figs. 2–6). In both analyzed MIH groups, an underabundance of proteins primarily associated with cell organization and biogenesis was identified. Additionally, proteins linked to cell death are predominantly overabundant in both MIH groups. Mukthar et al. (2022) reported proteins, found in MIH enamel to be mainly involved in biological processes with immune and inflammatory responses. In accordance with Mukthar et al*.* (2022), the present study found annexin A2, keratin type II cytoskeletal 1b, keratin type II cytoskeletal 78, plakophilin-1, and small proline-rich protein to be significantly overabundant in the MIH-affected enamel. In contrast to Mukthar et al. (2022), dentin sialophosphoprotein (DSPP), a protein transiently expressed during early enamel formation, was not identified in normal, or MIH enamel.

Several keratins were detected, some at high levels, e.g., keratins 16, 27 and 71 were found to be overabundant in MIH enamel. The occurrence of keratins in enamel has been questioned. Some keratins might play an important role in the formation of tooth enamel (Duverger et al. 2014; 2016; 2019). Given that keratins are cytoskeletal proteins, further studies need to reveal if they are deposited in enamel through exocytosis or with ameloblast fragments retained in enamel (Duverger et al. 2014, 2016 and 2019; Yang et al. 2019; Deshmukh et al. 2022).

Desmoplakin, desmoglein, and desmocollin were found to be overabundant in the MIH I enamel. Desmogleins and desmocollins, both transmembrane desmosomal cadherins, maintain extracellular cell–cell adhesion. Mutations in desmoplakin may lead to enamel abnormalities (Mahoney et al. 2010; Bartlett and Smith 2013). The role of proteinases in degrading junctional complexes, as well as the remodeling of junctional complexes during normal enamel developmental processes, has only been minimally investigated. Hypothesizing defective desmosome adhesion and degradation may affect amelogenesis (Bartlett and Smith 2013). The existence of enamel non-specific proteins in both normal and MIH enamel has been reported elsewhere (Açil et al. 2005; Farah et al. 2010; Jagr et al. 2019; Mukthar et al. 2022). Whether some of these proteins could constitute a possible source of contamination due to dissection and preparation of enamel samples for protein analysis, or constitute a part of the enamel proteome, is yet to be fully explained.

This study encounters similar limitations related to the microdissection of erupted permanent enamel, as discussed in previous studies (Jagr et al., 2019; Green et al. 2019; Gil-Bona and Bidlack 2020; Rexhaj et al. 2023). In the study conducted by Rexhaj et al. (2023), the challenges posed by the highly mineralized nature of dental enamel during microdissection and protein extraction, resulting in reduced protein content and limited accessibility due to low protein abundance, are highlighted. Furthermore, the proximity of dental tissues to enamel could potentially introduce contamination during sample dissection and preparation for protein analysis. While great care was taken during sample preparation to avoid contamination, it is important to note that some degree of contamination may still affect any proteomic dataset obtained from micro-sampled tissues.

To address potential contamination issues, all enamel surfaces underwent 35% phosphoric acid etching before microdissection, thereby eliminating surface debris. The increased porosity of MIH enamel may permit the adsorption of organic compounds from dietary sources, saliva, or pellicle onto the outermost enamel surface layer. The use of phosphoric acid, as a cleaning step, primarily affects a shallow layer of the outermost enamel surface, typically spanning a few micrometers. Importantly, the sample collection was obtained at a depth of 1–2 mm in the enamel, considered not to be affected using phosphoric acid as a detergent step (Torres-Rodríguez et al. 2012).

With the discovery of an increasing number of enamel matrix components across different studies, several questions appear: which proteins are endogenous, exogenous, or essential? and how do they contribute to enamel development (Gil-Bona and Bidlack 2020)? Another challenge and limitation is the lack of a standardized protocol for sample storage prior to microdissection and protein analysis, cleaning of teeth samples, microdissection, and use of the same methodological protocols for proteomic analyses. This complicates the comparison of data across different studies. By employing a combination of microdissection methodology, validated by Rexhaj et al. (2023), and LC–MS technique, this study showcases the capability to distinguish distinct proteomic profiles in MIH-affected teeth across various clinical severity grades. In this aspect, a strength of this study lies in the meticulous sampling and preparative protocols implemented, crucial for minimizing external contaminants. An inherent limitation in the study relates to the number of measurements conducted with the LC–MS, 11-plex equipment, which was constrained to 11 samples. With this sample size, the interpretation of the results must be approached with care, underscoring the need for further data collection to enhance reliability.

## Conclusions

Considering the limitations of the present in vitro study, it has been shown that there are variations in proteomic profiles within normal enamel and across different clinical severity grades of MIH enamel. This enables the possibility of using proteomic analysis to distinguish between distinct clinical severity grades of MIH. The potential applications of proteomics in advancing the understanding of this condition are promising and warrant further exploration in future investigations.

### Supplementary Information

Below is the link to the electronic supplementary material.

Supplementary file1 (DOCX 65 kb)
