# Supplementary material for: Proteomic profiling of human dental enamel affected by molar incisor hypomineralisation of different clinical severity grades: an in vitro study
Source: Eur Arch Paediatr Dent. 2024 Jun 6;25(4):533–45. doi: 10.1007/s40368-024-00911-9 (PMC11341683; doi:10.1007/s40368-024-00911-9)
Supplement: Supplementary file 1 — Supplementary file1 (DOCX 65 kb) [file 40368_2024_911_MOESM1_ESM.docx]

**MIH I + II Underabundance**

| Accession | Description | Biological Process |
| --- | --- | --- |
| P05107 | Integrin beta-2 | cell communication |
| P60953 | Cell division control protein 42 homolog | cell communication |
| P01019 | Angiotensinogen | cell communication |
| P11142 | Heat shock cognate 71 kDa protein | cell communication |
| P09382 | Galectin-1 | cell death |
| P23396 | 40S ribosomal protein S3 | cell death |
| P06396 | Gelsolin | cell death |
| P05109 | Protein S100-A8 | cell death |
| P98160 | Basement membrane-specific heparan sulfate proteoglycan core protein | cell differentiation |
| P26038 | Moesin | cell differentiation |
| P00747 | Plasminogen | cell differentiation |
| P24158 | Myeloblastin | cell differentiation |
| P04083 | Annexin A1 | cell differentiation |
| P02751 | Fibronectin | cell differentiation |
| P08670 | Vimentin | cell differentiation |
| O75083 | WD repeat-containing protein 1 | cell differentiation |
| P11215 | Integrin alpha-M | cell differentiation |
| P07910 | Heterogeneous nuclear ribonucleoproteins C1/C2 | cell differentiation |
| P62328 | Thymosin beta-4 | cell differentiation |
| P55145 | Mesencephalic astrocyte-derived neurotrophic factor | cell differentiation |
| P05186 | Alkaline phosphatase, tissue-nonspecific isozyme | cell differentiation |
| Q9HD89 | Resistin | cell differentiation |
| P02750 | Leucine-rich alpha-2-glycoprotein | cell differentiation |
| P62826 | GTP-binding nuclear protein Ran | cell division |
| Q05707 | Collagen alpha-1(XIV) chain | cell organization and biogenesis |
| P14174 | Macrophage migration inhibitory factor | cell organization and biogenesis |
| P02647 | Apolipoprotein A-I | cell organization and biogenesis |
| P61224 | Ras-related protein Rap-1b | cell organization and biogenesis |
| P00441 | Superoxide dismutase [Cu-Zn] | cell organization and biogenesis |
| P01857 | Immunoglobulin heavy constant gamma 1 | cell organization and biogenesis |
| P07996 | Thrombospondin-1 | cell organization and biogenesis |
| P60709 | Actin, cytoplasmic 1 | cell organization and biogenesis |
| P21333 | Filamin-A | cell organization and biogenesis |
| Q01518 | Adenylyl cyclase-associated protein 1 | cell organization and biogenesis |
| O15511 | Actin-related protein 2/3 complex subunit 5 | cell organization and biogenesis |
| Q9Y490 | Talin-1 | cell organization and biogenesis |
| P08133 | Annexin A6 | cell organization and biogenesis |
| P02671 | Fibrinogen alpha chain | cell organization and biogenesis |
| P02675 | Fibrinogen beta chain | cell organization and biogenesis |
| P68871 | Hemoglobin subunit beta | cell organization and biogenesis |
| P02679 | Fibrinogen gamma chain | cell organization and biogenesis |
| P13987 | CD59 glycoprotein | cell organization and biogenesis |
| P02748 | Complement component C9 | cell organization and biogenesis |
| P01861 | Immunoglobulin heavy constant gamma 4 | cell organization and biogenesis |
| P01859 | Immunoglobulin heavy constant gamma 2 | cell organization and biogenesis |
| P01860 | Immunoglobulin heavy constant gamma 3 | cell organization and biogenesis |
| P01024 | Complement C3 | cell organization and biogenesis |
| P06727 | Apolipoprotein A-IV | cell organization and biogenesis |
| Q15661 | Tryptase alpha/beta-1 | cell organization and biogenesis |
| P08697 | Alpha-2-antiplasmin | cell organization and biogenesis |
| P51888 | Prolargin | cell organization and biogenesis |
| P62310 | U6 snRNA-associated Sm-like protein LSm3 | cell organization and biogenesis |
| A8MVU1 | Putative neutrophil cytosol factor 1C | cell organization and biogenesis |
| Q04837 | Single-stranded DNA-binding protein, mitochondrial | cell organization and biogenesis |
| P16402 | Histone H1.3 | cell organization and biogenesis |
| P50454 | Serpin H1 | cell organization and biogenesis |
| P51884 | Lumican | cell organization and biogenesis |
| P07585 | Decorin | cell organization and biogenesis |
| P23142 | Fibulin-1 | cell organization and biogenesis |
| P39060 | Collagen alpha-1(XVIII) chain | cell organization and biogenesis |
| O95445 | Apolipoprotein M | cell organization and biogenesis |
| P69905 | Hemoglobin subunit alpha | cell organization and biogenesis |
| P02656 | Apolipoprotein C-III | cell organization and biogenesis |
| P02655 | Apolipoprotein C-II OS | cell organization and biogenesis |
| P02766 | Transthyretin | cell organization and biogenesis |
| P02654 | Apolipoprotein C-I | cell organization and biogenesis |
| P62277 | 40S ribosomal protein S13 | cell organization and biogenesis |
| P28676 | Grancalcin | cell organization and biogenesis |
| O75367 | Core histone macro-H2A.1 | cell organization and biogenesis |
| P40121 | Macrophage-capping protein | cell organization and biogenesis |
| P29966 | Myristoylated alanine-rich C-kinase substrate | cell organization and biogenesis |
| P26447 | Protein S100-A4 | cell organization and biogenesis |
| P07737 | Profilin-1 | cell organization and biogenesis |
| P59998 | Actin-related protein 2/3 complex subunit 4 | cell organization and biogenesis |
| P08514 | Integrin alpha-Iib | cell organization and biogenesis |
| P06703 | Protein S100-A6 | cell organization and biogenesis |
| Q15286 | Ras-related protein Rab-35 | cell organization and biogenesis |
| P49458 | Signal recognition particle 9 kDa protein | cell organization and biogenesis |
| P09497 | Clathrin light chain B | cell organization and biogenesis |
| P80511 | Protein S100-A12 | cellular component movement |
| P20160 | Azurocidin | cellular component movement |
| P41218 | Myeloid cell nuclear differentiation antigen | cellular component movement |
| P02775 | Platelet basic protein | cellular component movement |
| P35542 | Serum amyloid A-4 protein | cellular component movement |
| P52566 | Rho GDP-dissociation inhibitor 2 | cellular component movement |
| P20340 | Ras-related protein Rab-6A | cellular component movement |
| P52565 | Rho GDP-dissociation inhibitor 1 | cellular component movement |
| P01042 | Kininogen-1 | cellular homeostasis |
| P00390 | Glutathione reductase, mitochondrial | cellular homeostasis |
| P00450 | Ceruloplasmin | cellular homeostasis |
| O75368 | SH3 domain-binding glutamic acid-rich-like protein | cellular homeostasis |
| P02042 | Hemoglobin subunit delta | coagulation |
| P07357 | Complement component C8 alpha chain | defense response |
| P02747 | Complement C1q subcomponent subunit C | defense response |
| P0C0L4 | Complement C4-A | defense response |
| P00751 | Complement factor B | defense response |
| P07360 | Complement component C8 gamma chain | defense response |
| P07358 | Complement component C8 beta chain | defense response |
| Q14624 | Inter-alpha-trypsin inhibitor heavy chain H4 | defense response |
| P00738 | Haptoglobin | defense response |
| P59666 | Neutrophil defensin 3 | defense response |
| P12429 | Annexin A3 | defense response |
| P19652 | Alpha-1-acid glycoprotein 2 | defense response |
| P02763 | Alpha-1-acid glycoprotein 1 | defense response |
| P49913 | Cathelicidin antimicrobial peptide | defense response |
| P60174 | Triosephosphate isomerase | development |
| Q15717 | ELAV-like protein 1 | development |
| P04843 | Dolichyl-diphosphooligosaccharide--protein glycosyltransferase subunit 1 | metabolic process |
| Q13011 | Delta(3,5)-Delta(2,4)-dienoyl-CoA isomerase, mitochondrial | metabolic process |
| P07311 | Acylphosphatase-1 | metabolic process |
| Q9HDC9 | Adipocyte plasma membrane-associated protein | metabolic process |
| P30046 | D-dopachrome decarboxylase | metabolic process |
| P23284 | Peptidyl-prolyl cis-trans isomerase B | metabolic process |
| Q9UKM9 | RNA-binding protein Raly | metabolic process |
| P19823 | Inter-alpha-trypsin inhibitor heavy chain H2 | metabolic process |
| P29401 | Transketolase | metabolic process |
| Q8TAX7 | Mucin-7 | metabolic process |
| P61978 | Heterogeneous nuclear ribonucleoprotein K | metabolic process |
| Q14103 | Heterogeneous nuclear ribonucleoprotein D0 | metabolic process |
| P06733 | Alpha-enolase | metabolic process |
| P61204 | ADP-ribosylation factor 3 | metabolic process |
| P00915 | Carbonic anhydrase 1 | metabolic process |
| P30040 | Endoplasmic reticulum resident protein 29 | metabolic process |
| P22314 | Ubiquitin-like modifier-activating enzyme 1 | metabolic process |
| Q5JXB2 | Putative ubiquitin-conjugating enzyme E2 N-like | metabolic process |
| O00754 | Lysosomal alpha-mannosidase | metabolic process |
| P30043 | Flavin reductase (NADPH) | metabolic process |
| P05534 | HLA class I histocompatibility antigen, A-24 alpha chain | regulation of biological process |
| P63218 | Guanine nucleotide-binding protein G(I)/G(S)/G(O) subunit gamma-5 | regulation of biological process |
| P19338 | Nucleolin | regulation of biological process |
| Q9UHF0 | Tachykinin-3 | regulation of biological process |
| P04080 | Cystatin-B | regulation of biological process |
| P05452 | Tetranectin | regulation of biological process |
| P31949 | Protein S100-A11 | regulation of biological process |
| Q99584 | Protein S100-A13 | regulation of biological process |
| P51148 | Ras-related protein Rab-5C | regulation of biological process |
| P04217 | Alpha-1B-glycoprotein | transport |

**MIH I + II Overabundance**

| Accession | Description | Biological Process |
| --- | --- | --- |
| P02452 | Collagen alpha-1(I) chain OS | cell communication |
| Q8N1N4 | Keratin, type II cytoskeletal 78 | cell death |
| Q9C075 | Keratin, type I cytoskeletal 23 | cell death |
| Q9NSB4 | Keratin, type II cuticular Hb2 | cell death |
| Q7Z3Y8 | Keratin, type I cytoskeletal 27 | cell death |
| P01137 | Transforming growth factor beta-1 proprotein | cell death |
| P08779 | Keratin, type I cytoskeletal 16 | cell death |
| P22735 | Protein-glutamine gamma-glutamyltransferase K | cell death |
| P15924 | Desmoplakin | cell death |
| P05787 | Keratin, type II cytoskeletal 8 | cell death |
| P22528 | Cornifin-B | cell death |
| P13645 | Keratin, type I cytoskeletal 10 | cell death |
| P22532 | Small proline-rich protein 2D | cell death |
| Q15517 | Corneodesmosin | cell death |
| Q7Z794 | Keratin, type II cytoskeletal 1b | cell death |
| P14923 | Junction plakoglobin | cell death |
| P13647 | Keratin, type II cytoskeletal 5 | cell death |
| P04259 | Keratin, type II cytoskeletal 6B | cell death |
| Q9HCY8 | Protein S100-A14 | cell death |
| O76011 | Keratin, type I cuticular Ha4 | cell death |
| Q6KB66 | Keratin, type II cytoskeletal 80 | cell death |
| Q08554 | Desmocollin-1 | cell death |
| Q14533 | Keratin, type II cuticular Hb1 | cell death |
| Q5T750 | Skin-specific protein 32 | cell differentiation |
| Q8WVV4 | Protein POF1B | cell organization and biogenesis |
| P06753 | Tropomyosin alpha-3 chain | cell organization and biogenesis |
| O15144 | Actin-related protein 2/3 complex subunit 2 | cell organization and biogenesis |
| Q86YZ3 | Hornerin | cell organization and biogenesis |
| P07477 | Trypsin-1 | cell organization and biogenesis |
| P48163 | NADP-dependent malic enzyme | cell organization and biogenesis |
| P61254 | 60S ribosomal protein L26 | cell organization and biogenesis |
| P01619 | Immunoglobulin kappa variable 3-20 | cellular component movement |
| P69892 | Hemoglobin subunit gamma-2 | coagulation |
| Q04118 | Basic salivary proline-rich protein 3 | defense response |
| Q8NEX9 | Short-chain dehydrogenase/reductase family 9C member 7 | metabolic process |
| P25789 | Proteasome subunit alpha type-4 | metabolic process |
| P28066 | Proteasome subunit alpha type-5 | metabolic process |
| P28072 | Proteasome subunit beta type-6 | metabolic process |
| Q6ZVX7 | F-box only protein 50 | regulation of biological process |
| O75556 | Mammaglobin-B | regulation of biological process |
| P01036 | Cystatin-S | regulation of biological process |
| Q5D862 | Filaggrin-2 | transport |
| Q5T749 | Keratinocyte proline-rich protein | no data |

|  | **MIH I Underabundance** |  |
| --- | --- | --- |
| **Accession** | **Description** | **Biological Process** |
| P05107 | Integrin beta-2 | cell communication |
| P11142 | Heat shock cognate 71 kDa protein | cell communication |
| P08727 | Keratin, type I cytoskeletal 19 | cell death |
| P09382 | Galectin-1 | cell death |
| P23396 | 40S ribosomal protein S3 | cell death |
| P06396 | Gelsolin | cell death |
| P61586 | Transforming protein RhoA | cell differentiation |
| P26038 | Moesin | cell differentiation |
| P00747 | Plasminogen | cell differentiation |
| P24158 | Myeloblastin | cell differentiation |
| P02751 | Fibronectin | cell differentiation |
| P13611 | Versican core protein | cell differentiation |
| P08670 | Vimentin | cell differentiation |
| O75083 | WD repeat-containing protein 1 | cell differentiation |
| P07910 | Heterogeneous nuclear ribonucleoproteins C1/C2 | cell differentiation |
| P22894 | Neutrophil collagenase | cell differentiation |
| P61604 | 10 kDa heat shock protein, mitochondrial | cell differentiation |
| P02750 | Leucine-rich alpha-2-glycoprotein | cell differentiation |
| P61160 | Actin-related protein 2 | cell division |
| P62826 | GTP-binding nuclear protein Ran | cell division |
| P02647 | Apolipoprotein A-I | cell organization and biogenesis |
| P62750 | 60S ribosomal protein L23a | cell organization and biogenesis |
| P61224 | Ras-related protein Rap-1b | cell organization and biogenesis |
| P62820 | Ras-related protein Rab-1A | cell organization and biogenesis |
| P01857 | Immunoglobulin heavy constant gamma 1 | cell organization and biogenesis |
| P01876 | Immunoglobulin heavy constant alpha 1 | cell organization and biogenesis |
| P60709 | Actin, cytoplasmic 1 | cell organization and biogenesis |
| P62937 | Peptidyl-prolyl cis-trans isomerase A | cell organization and biogenesis |
| P21333 | Filamin-A | cell organization and biogenesis |
| Q9Y490 | Talin-1 | cell organization and biogenesis |
| Q01518 | Adenylyl cyclase-associated protein 1 | cell organization and biogenesis |
| P22392 | Nucleoside diphosphate kinase B | cell organization and biogenesis |
| Q9UJZ1 | Stomatin-like protein 2, mitochondrial | cell organization and biogenesis |
| P08133 | Annexin A6 | cell organization and biogenesis |
| P02671 | Fibrinogen alpha chain | cell organization and biogenesis |
| P02675 | Fibrinogen beta chain | cell organization and biogenesis |
| P68871 | Hemoglobin subunit beta | cell organization and biogenesis |
| P02679 | Fibrinogen gamma chain | cell organization and biogenesis |
| P01860 | Immunoglobulin heavy constant gamma 3 | cell organization and biogenesis |
| P01861 | Immunoglobulin heavy constant gamma 4 | cell organization and biogenesis |
| P01859 | Immunoglobulin heavy constant gamma 2 | cell organization and biogenesis |
| P01024 | Complement C3 | cell organization and biogenesis |
| P06727 | Apolipoprotein A-IV | cell organization and biogenesis |
| Q15661 | Tryptase alpha/beta-1 | cell organization and biogenesis |
| P62888 | 60S ribosomal protein L30 | cell organization and biogenesis |
| P08697 | Alpha-2-antiplasmin | cell organization and biogenesis |
| P07305 | Histone H1.0 | cell organization and biogenesis |
| P51888 | Prolargin | cell organization and biogenesis |
| A8MVU1 | Putative neutrophil cytosol factor 1C | cell organization and biogenesis |
| Q04837 | Single-stranded DNA-binding protein, mitochondrial | cell organization and biogenesis |
| P16402 | Histone H1.3 | cell organization and biogenesis |
| P50454 | Serpin H1 | cell organization and biogenesis |
| P39060 | Collagen alpha-1(XVIII) chain O | cell organization and biogenesis |
| P16104 | Histone H2AX | cell organization and biogenesis |
| P51884 | Lumican | cell organization and biogenesis |
| P07585 | Decorin | cell organization and biogenesis |
| P62805 | Histone H4 | cell organization and biogenesis |
| P23142 | Fibulin-1 | cell organization and biogenesis |
| P69905 | Hemoglobin subunit alpha | cell organization and biogenesis |
| O95445 | Apolipoprotein M | cell organization and biogenesis |
| P02656 | Apolipoprotein C-III | cell organization and biogenesis |
| P02655 | Apolipoprotein C-II | cell organization and biogenesis |
| P62244 | 40S ribosomal protein S15a | cell organization and biogenesis |
| P62277 | 40S ribosomal protein S13 | cell organization and biogenesis |
| P02654 | Apolipoprotein C-I | cell organization and biogenesis |
| P62913 | 60S ribosomal protein L11 | cell organization and biogenesis |
| P63220 | 40S ribosomal protein S21 | cell organization and biogenesis |
| P08865 | 40S ribosomal protein SA | cell organization and biogenesis |
| P18077 | 60S ribosomal protein L35a | cell organization and biogenesis |
| P62424 | 60S ribosomal protein L7a | cell organization and biogenesis |
| P62851 | 40S ribosomal protein S25 | cell organization and biogenesis |
| P46783 | 40S ribosomal protein S10 | cell organization and biogenesis |
| P26373 | 60S ribosomal protein L13 | cell organization and biogenesis |
| P29966 | Myristoylated alanine-rich C-kinase substrate | cell organization and biogenesis |
| P26447 | Protein S100-A4 | cell organization and biogenesis |
| P40121 | Macrophage-capping protein | cell organization and biogenesis |
| P59998 | Actin-related protein 2/3 complex subunit 4 | cell organization and biogenesis |
| P07737 | Profilin-1 | cell organization and biogenesis |
| Q15286 | Ras-related protein Rab-35 | cell organization and biogenesis |
| O15400 | Syntaxin-7 | cell organization and biogenesis |
| P49458 | Signal recognition particle 9 kDa protein | cell organization and biogenesis |
| P09497 | Clathrin light chain B | cell organization and biogenesis |
| P41218 | Myeloid cell nuclear differentiation antigen | cellular component movement |
| P02775 | Platelet basic protein | cellular component movement |
| P35542 | Serum amyloid A-4 protein | cellular component movement |
| P52566 | Rho GDP-dissociation inhibitor 2 | cellular component movement |
| P20340 | Ras-related protein Rab-6A | cellular component movement |
| P01042 | Kininogen-1 | cellular homeostasis |
| P00390 | Glutathione reductase | cellular homeostasis |
| P00450 | Ceruloplasmin | cellular homeostasis |
| O75368 | SH3 domain-binding glutamic acid-rich-like protein | cellular homeostasis |
| P02042 | Hemoglobin subunit delta | coagulation |
| P07357 | Complement component C8 alpha chain | defense response |
| P07358 | Complement component C8 beta chain | defense response |
| P0C0L4 | Complement C4-A | defense response |
| P13671 | Complement component C6 | defense response |
| P00751 | Complement factor B | defense response |
| P00738 | Haptoglobin | defense response |
| O75594 | Peptidoglycan recognition protein 1 | defense response |
| Q14624 | Inter-alpha-trypsin inhibitor heavy chain H4 | defense response |
| P61626 | Lysozyme C | defense response |
| P12429 | Annexin A3 | defense response |
| P19652 | Alpha-1-acid glycoprotein 2 | defense response |
| P02763 | Alpha-1-acid glycoprotein 1 | defense response |
| P49913 | Cathelicidin antimicrobial peptide | defense response |
| P60174 | Triosephosphate isomerase | development |
| Q15717 | ELAV-like protein 1 | development |
| P13929 | Beta-enolase | metabolic process |
| P24666 | Low molecular weight phosphotyrosine protein phosphatase | metabolic process |
| P04843 | Dolichyl-diphosphooligosaccharide--protein glycosyltransferase subunit 1 | metabolic process |
| P07311 | Acylphosphatase-1 | metabolic process |
| Q9HDC9 | Adipocyte plasma membrane-associated protein | metabolic process |
| P19823 | Inter-alpha-trypsin inhibitor heavy chain H2 | metabolic process |
| Q9UKM9 | RNA-binding protein Raly | metabolic process |
| P29401 | Transketolase | metabolic process |
| P23284 | Peptidyl-prolyl cis-trans isomerase B | metabolic process |
| P30086 | Phosphatidylethanolamine-binding protein 1 | metabolic process |
| P06733 | Alpha-enolase | metabolic process |
| Q8TAX7 | Mucin-7 | metabolic process |
| Q14103 | Heterogeneous nuclear ribonucleoprotein D0 | metabolic process |
| P61978 | Heterogeneous nuclear ribonucleoprotein K | metabolic process |
| Q9HAV0 | Guanine nucleotide-binding protein subunit beta-4 | metabolic process |
| P00915 | Carbonic anhydrase 1 | metabolic process |
| P22314 | Ubiquitin-like modifier-activating enzyme 1 | metabolic process |
| Q5JXB2 | Putative ubiquitin-conjugating enzyme E2 N-like | metabolic process |
| Q9Y3C8 | Ubiquitin-fold modifier-conjugating enzyme 1 | metabolic process |
| P30043 | Flavin reductase (NADPH) | metabolic process |
| O00754 | Lysosomal alpha-mannosidase | metabolic process |
| P63218 | Guanine nucleotide-binding protein G(I)/G(S)/G(O) subunit gamma-5 | regulation of biological process |
| Q9UHF0 | Tachykinin-3 | regulation of biological process |
| Q5HYI8 | Rab-like protein 3 | regulation of biological process |
| P05534 | HLA class I histocompatibility antigen, A-24 alpha chain | regulation of biological process |
| P51148 | Ras-related protein Rab-5C | regulation of biological process |
| P05452 | Tetranectin | regulation of biological process |
| P31949 | Protein S100-A11 | regulation of biological process |
| Q96JT2 | Solute carrier family 45 member 3 | regulation of biological process |
| P04217 | Alpha-1B-glycoprotein | transport |

|  | **MIH I Overabundance** |  |
| --- | --- | --- |
| **Accession** | **Description** | **Biological Process** |
| P04062 | Lysosomal acid glucosylceramidase | cell communication |
| Q8N1N4 | Keratin, type II cytoskeletal 78 | cell death |
| Q7Z3Y8 | Keratin, type I cytoskeletal 27 | cell death |
| Q14CN4 | Keratin, type II cytoskeletal 72 | cell death |
| Q9C075 | Keratin, type I cytoskeletal 23 | cell death |
| P08779 | Keratin, type I cytoskeletal 16 | cell death |
| P22735 | Protein-glutamine gamma-glutamyltransferase K | cell death |
| P15924 | Desmoplakin | cell death |
| P22528 | Cornifin-B | cell death |
| P13645 | Keratin, type I cytoskeletal 10 | cell death |
| P22532 | Small proline-rich protein 2D | cell death |
| Q15517 | Corneodesmosin | cell death |
| Q3SY84 | Keratin, type II cytoskeletal 71 | cell death |
| Q15323 | Keratin, type I cuticular Ha1 | cell death |
| Q7Z794 | Keratin, type II cytoskeletal 1b | cell death |
| P35908 | Keratin, type II cytoskeletal 2 epidermal | cell death |
| P14923 | Junction plakoglobin | cell death |
| Q13835 | Plakophilin-1 | cell death |
| P13647 | Keratin, type II cytoskeletal 5 | cell death |
| Q02413 | Desmoglein-1 | cell death |
| Q9HCY8 | Protein S100-A14 | cell death |
| Q6KB66 | Keratin, type II cytoskeletal 80 | cell death |
| Q14533 | Keratin, type II cuticular Hb1 | cell death |
| Q08554 | Desmocollin-1 | cell death |
| Q5T750 | Skin-specific protein 32 | cell differentiation |
| P49748 | Very long-chain specific acyl-CoA dehydrogenase, mitochondrial | cell differentiation |
| Q8WVV4 | Protein POF1B | cell organization and biogenesis |
| Q86YZ3 | Hornerin | cell organization and biogenesis |
| P07477 | Trypsin-1 | cell organization and biogenesis |
| P48163 | NADP-dependent malic enzyme | cell organization and biogenesis |
| P35555 | Fibrillin-1 | cell organization and biogenesis |
| P07355 | Annexin A2 | cell organization and biogenesis |
| P69892 | Hemoglobin subunit gamma-2 | coagulation |
| P81605 | Dermcidin | defense response |
| Q16610 | Extracellular matrix protein 1 | defense response |
| Q8NEX9 | Short-chain dehydrogenase | metabolic process |
| P25789 | Proteasome subunit alpha type-4 | metabolic process |
| P28072 | Proteasome subunit beta type-6 | metabolic process |
| Q01469 | Fatty acid-binding protein 5 | metabolic process |
| P01036 | Cystatin-S | regulation of biological process |
| Q5D862 | Filaggrin-2 | transport |
| Q5T749 | Keratinocyte proline-rich protein | no data |

|  | **MIH II Underabundance** |  |
| --- | --- | --- |
| **Accession** | **Description** | **Biological Process** |
| P05107 | Integrin beta-2 | cell communication |
| P09382 | Galectin-1 | cell death |
| P06396 | Gelsolin | cell death |
| P05109 | Protein S100-A8 | cell death |
| P00747 | Plasminogen | cell differentiation |
| P02751 | Fibronectin | cell differentiation |
| P11215 | Integrin alpha-M | cell differentiation |
| P07910 | Heterogeneous nuclear ribonucleoproteins C1/C2 | cell differentiation |
| P62328 | Thymosin beta-4 | cell differentiation |
| Q15149 | Plectin | cell organization and biogenesis |
| P14174 | Macrophage migration inhibitory factor | cell organization and biogenesis |
| P02647 | Apolipoprotein A-I | cell organization and biogenesis |
| P00441 | Superoxide dismutase [Cu-Zn] | cell organization and biogenesis |
| P07996 | Thrombospondin-1 | cell organization and biogenesis |
| Q9Y490 | Talin-1 | cell organization and biogenesis |
| P02671 | Fibrinogen alpha chain | cell organization and biogenesis |
| P02679 | Fibrinogen gamma chain | cell organization and biogenesis |
| P02743 | Serum amyloid P-component | cell organization and biogenesis |
| P01859 | Immunoglobulin heavy constant gamma 2 | cell organization and biogenesis |
| P02748 | Complement component C9 | cell organization and biogenesis |
| P06727 | Apolipoprotein A-IV | cell organization and biogenesis |
| P01024 | Complement C3 | cell organization and biogenesis |
| P51884 | Lumican | cell organization and biogenesis |
| P07585 | Decorin | cell organization and biogenesis |
| P23142 | Fibulin-1 | cell organization and biogenesis |
| O95445 | Apolipoprotein M | cell organization and biogenesis |
| P28676 | Grancalcin | cell organization and biogenesis |
| P26447 | Protein S100-A4 | cell organization and biogenesis |
| P29966 | Myristoylated alanine-rich C-kinase substrate | cell organization and biogenesis |
| P08514 | Integrin alpha-Iib | cell organization and biogenesis |
| P06703 | Protein S100-A6 | cell organization and biogenesis |
| P80511 | Protein S100-A12 | cellular component movement |
| P41218 | Myeloid cell nuclear differentiation antigen | cellular component movement |
| P02775 | Platelet basic protein | cellular component movement |
| P52565 | Rho GDP-dissociation inhibitor 1 | cellular component movement |
| P00450 | Ceruloplasmin | cellular homeostasis |
| P07357 | Complement component C8 alpha chain | defense response |
| P0C0L4 | Complement C4-A | defense response |
| Q14624 | Inter-alpha-trypsin inhibitor heavy chain H4 | defense response |
| P59666 | Neutrophil defensin 3 | defense response |
| P02741 | C-reactive protein | defense response |
| P19652 | Alpha-1-acid glycoprotein 2 | defense response |
| P02763 | Alpha-1-acid glycoprotein 1 | defense response |
| P12429 | Annexin A3 | defense response |
| P49913 | Cathelicidin antimicrobial peptide | defense response |
| P60174 | Triosephosphate isomerase | development |
| Q07065 | Cytoskeleton-associated protein 4 | metabolic process |
| Q9UHF0 | Tachykinin-3 | regulation of biological process |
| P19338 | Nucleolin | regulation of biological process |
| P05452 | Tetranectin | regulation of biological process |
| Q99584 | Protein S100-A13 | regulation of biological process |
| P04080 | Cystatin-B | regulation of biological process |
| P31949 | Protein S100-A11 | regulation of biological process |

|  | **MIH II Overabundance** |  |
| --- | --- | --- |
| **Accession** | **Description** | **Biological Process** |
| P05787 | Keratin, type II cytoskeletal 8 | cell death |
| P14923 | Junction plakoglobin | cell death |
| Q08554 | Desmocollin-1 | cell death |
| Q9UHD8 | Septin-9 | cell division |
| P06753 | Tropomyosin alpha-3 chain | cell organization and biogenesis |
| Q04917 | 14-3-3 protein eta | cell organization and biogenesis |
| P28066 | Proteasome subunit alpha type-5 | metabolic process |
